# Supplementary material for: Automating candidate gene prioritization with large language models: from naive scoring to literature-grounded validation
Source: Bioinformatics. 2025 Oct 10;41(10):btaf541. doi: 10.1093/bioinformatics/btaf541 (PMC12548045; doi:10.1093/bioinformatics/btaf541)
Supplement: btaf541_Supplementary_Data [file btaf541_supplementary_data.zip › SupplementaryMaterial_2_Comprehensive Evaluation of 30 Sepsis-Associated Genes (1).pdf]

ChatGPT

Comprehensive Evaluation of 30 Sepsis-Associated Genes

Introduction

Sepsis is a complex syndrome involving dysregulated immune responses, coagulopathy, and organ dysfunction. Numerous genes contribute to its pathophysiology, spanning innate immune receptors, inflammatory mediators, metabolic regulators, and endothelial factors. Below, we evaluate 30 prioritized genes for their roles in sepsis pathophysiology, therapeutic relevance, biomarker potential, expression patterns in circulating cells, and the novel insights they offer beyond standard sepsis markers (e.g. classic cytokines like TNF or IL-6).

CXCR2 (C-X-C Chemokine Receptor 2)

- **Role in Sepsis:** CXCR2 is a chemokine receptor on neutrophils that mediates their recruitment to infection sites in response to ELR⁺ CXC chemokines (e.g. IL-8/CXCL8). In severe sepsis, systemic TLR agonists trigger GRK2-dependent internalization of CXCR2 on neutrophils, blunting chemotaxis despite high chemokine levels. This impaired migration contributes to uncontrolled infection and allows neutrophils to mislocalize to distant organs (via upregulated CCR2), causing collateral tissue damage.
- **Therapeutic Potential:** Restoring proper CXCR2 function or blocking its aberrant signals has dual implications. Augmenting CXCR2 signaling can improve neutrophil recruitment to infections; for example, IL-33 treatment prevented CXCR2 internalization and improved neutrophil trafficking in models. Conversely, targeted CXCR2 inhibition may protect vulnerable organs: in an LPS sepsis encephalopathy model, a CXCR2 antagonist reduced neutrophil brain infiltration and preserved blood–brain barrier integrity. Such context-specific modulation of CXCR2 could temper harmful neutrophil activity without broadly immunosuppressing the host.
- **Biomarker Potential:** Neutrophil surface CXCR2 levels appear as a sepsis-specific diagnostic indicator. At disease onset, septic patients show a marked CXCR2 downregulation (inversely correlated with severity), distinguishing sepsis from non-septic infection [1]. Persistent low CXCR2 is associated with worse outcomes, whereas survivors tend to maintain higher CXCR2 and better chemotaxis. Thus, flow-cytometric CXCR2 measurement on neutrophils has been proposed to identify septic patients and predict prognosis.
- **Expression in Blood Cells:** CXCR2 is predominantly expressed on neutrophils (and to a lesser extent on monocytes and endothelial cells). During non-severe infection, neutrophils with surface CXCR2 efficiently migrate to tissues. In sepsis, circulating neutrophils rapidly lose surface CXCR2 via internalization. Endothelial CXCR2 also plays a role in transendothelial neutrophil migration, particularly in the brain microvasculature.
- **Novel Insights:** CXCR2 highlights an underappreciated aspect of sepsis: **neutrophil trafficking dysregulation**. While classic sepsis paradigms focus on cytokine storms, CXCR2's behavior reveals how sepsis induces immune **paralysis and misdirection** – neutrophils become less responsive to infection foci yet cause organ damage elsewhere [1]. Targeting this chemokine axis offers a more nuanced approach than broad anti-inflammatories, aiming to restore host defense while limiting collateral injury.

PTX3 (Pentraxin 3)

- **Role in Sepsis:** PTX3 is a long pentraxin released by innate immune cells (monocytes, macrophages, dendritic cells) and endothelial cells in response to primary inflammatory signals (TLRs, IL-1, TNF). Unlike short pentraxins (e.g. CRP from liver), PTX3 is produced at infection sites and acts as a soluble pattern-recognition molecule. It binds microbial motifs, opsonizes pathogens, and modulates complement activation and neutrophil recruitment. In sepsis, PTX3 levels rise rapidly and correlate with disease severity and survival. Functionally, PTX3 has a dual role: it can limit excessive inflammation by binding P-selectin and dampening neutrophil extravasation, while also enhancing host defense – PTX3-deficient mice exhibit impaired clearance of bacteria and fungi, whereas administering recombinant PTX3 improves pathogen elimination [2]. Notably, PTX3 neutralizes certain DAMPs (e.g. histones), reducing their toxicity.
- **Therapeutic Potential:** As an endogenous immune regulator, PTX3 is being explored both as a **therapeutic agent** and target. In preclinical models, **supplementing PTX3** (alone or with antimicrobials) protected against septic challenges – for instance, exogenous PTX3 improved survival in fungal sepsis and in polymicrobial peritonitis by enhancing microbial clearance [2]. These findings suggest that boosting PTX3 could bolster humoral innate immunity in sepsis. Conversely, extremely high PTX3 levels may contribute to inflammation; thus, titrating its activity might be key. No specific PTX3 inhibitors are in use – rather, its **beneficial effects** (facilitating complement and preventing leukocyte over-recruitment) are what make PTX3 an attractive adjunct therapy candidate.
- **Biomarker Potential:** PTX3 has emerged as a robust **biomarker of sepsis severity and outcome**. Circulating PTX3 concentrations are significantly higher in septic patients (and especially non-survivors) compared to controls, and levels track with organ failure scores. Importantly, PTX3 rises earlier than CRP during infection, reflecting rapid local immune activation. Meta-analyses confirm that PTX3 plasma levels strongly predict 28-day mortality and can stratify patients by risk. Its performance as a prognostic marker is on par with or complementary to IL-6. These qualities position PTX3 as a valuable addition to sepsis biomarker panels for early risk assessment.
- **Expression in Circulating Cells:** PTX3 is stored in specific granules of neutrophils and is readily secreted upon activation. Monocytes, macrophages, dendritic cells, and endothelial cells all produce PTX3 in response to inflammatory stimuli. (Notably, lymphocytes do not express PTX3 [3].) In sepsis, blood neutrophils release pre-formed PTX3 immediately during the early inflammatory surge, and mononuclear phagocytes sustain PTX3 production during ongoing infection. This broad cellular source ensures that PTX3 levels in blood mirror the activation of the innate immune compartment across the body.
- **Novel Insights:** PTX3 provides a **humoral innate immunity** perspective distinct from classic pro-inflammatory cytokines. It underscores mechanisms of pathogen recognition and containment (complement activation, extracellular traps) not captured by measuring TNF or IL-6. Clinically, whereas CRP is an indirect acute-phase reactant, PTX3 directly reflects tissue-level immune responses and endothelial involvement. It thus adds a **novel axis of information** – bridging inflammation and coagulation – and its successful therapeutic use in models suggests sepsis treatment could go beyond blocking inflammation to **enhancing protective innate opsonins** [2].

SIRT1 (Sirtuin 1)

- **Role in Sepsis:** SIRT1 is an NAD⁺-dependent deacetylase that acts as a master regulator of inflammation, metabolism, and stress responses. In immune cells, SIRT1 deacetylates the NF-κB p65 subunit, blunting NF-κB's transcription of pro-inflammatory cytokines. It also deacetylates factors like FOXO3a and p53, promoting antioxidant defenses and

mitochondrial biogenesis [4]. During sepsis, SIRT1 expression/activity typically **decreases** (e.g. septic patients show lower SIRT1 levels than healthy controls) [5], which removes a check on unchecked inflammation. Low SIRT1 is associated with higher circulating TNF, IL-6, and adhesion molecules [5], exacerbating tissue injury. Paradoxically, SIRT1's anti-inflammatory effect can contribute to immune suppression: it facilitates M2 macrophage polarization and Treg generation. In late sepsis, high SIRT1 in dendritic cells/T cells may suppress critical immune responses. Overall, SIRT1 helps maintain balance – curbing the early cytokine storm and protecting endothelium [6], while supporting metabolic adaptation (switch to fatty acid oxidation via PGC-1α) in prolonged sepsis.
- **Therapeutic Potential:** Enhancing SIRT1 activity is a promising strategy to mitigate organ damage in sepsis. **SIRT1 activators** (e.g. SRT1720) significantly reduced organ injury and inflammation in septic mice: treated animals had lower AST/ALT (less liver injury), reduced IL-1β/IL-6 levels, and suppressed NLRP3 inflammasome activation. SRT1720-treated septic mice experienced attenuated multi-organ failure and improved survival, highlighting that pharmacological **stimulation of SIRT1** can dampen cytokine release and inflammasome-driven damage. Clinically, strategies like resveratrol (a SIRT1 activator) or small molecules are under exploration to replicate these benefits. Additionally, SIRT1 upregulation (e.g. by IFN-β) in immune cells has been shown to restore bacterial clearance in septic models. Notably, care must be taken in timing – excessive SIRT1 activation in late sepsis could reinforce immunosuppression. Nonetheless, **bolstering SIRT1** early to mid-course appears to confer a cytoprotective, inflammation-resolving effect without completely shutting down host defense.
- **Biomarker Potential:** SIRT1 itself can be considered an indicator of immune homeostasis in sepsis. *Lower* SIRT1 levels in patients correlate with greater disease severity (inversely related to APACHE II/SOFA scores) and worse survival [5]. In one clinical study, non-survivors had significantly depressed SIRT1 compared to survivors during the first week of ICU stay [7]. Thus, **serum SIRT1 levels** (or monocyte SIRT1 activity) might serve as a marker of immunoparalysis and risk of poor outcome. Indeed, SIRT1 level was found to distinguish septic patients from healthy controls with reasonable sensitivity [5]. As therapies that boost SIRT1 are developed, its level could also guide patient selection for such immunometabolic interventions.
- **Expression in Circulating Cells:** SIRT1 is ubiquitously expressed, with high levels in the nucleus of monocytes, lymphocytes, and endothelial cells. It shuttles to the cytoplasm under stress conditions [8]. In sepsis, circulating immune cells (e.g. PBMCs) show reduced SIRT1 expression/activity, which is linked to increased acetylation of NF-κB and heightened cytokine output [5]. SIRT1 in endothelium is also crucial: it deacetylates proteins to maintain tight junctions and reduce permeability – SIRT1 activation has been shown to **decrease vascular leak** via effects on cytoskeletal regulators (e.g. RhoA/ROCK). Overall, SIRT1's presence in blood leukocytes and endothelium makes it a central mediator of the systemic response.
- **Novel Insights:** SIRT1 exemplifies the **epigenetic and metabolic regulation** of sepsis. It links energy status (NAD⁺ levels) to inflammation, a dimension not addressed by standard cytokine biomarkers. Its decline in sepsis provides a mechanism for excessive inflammation and organ damage via unchecked NF-κB signaling. Conversely, SIRT1's role in promoting **immune cell survival and metabolic flexibility** offers a new therapeutic angle: instead of neutralizing a single cytokine, modulating SIRT1 can broadly recalibrate the immune response to be both effective against microbes and less damaging to the host. This positions SIRT1 at the crossroads of immunity and metabolism, an axis that standard therapies have yet to exploit.

C5 (Complement Component 5)

- **Role in Sepsis:** C5 is a central complement protein cleaved into the potent anaphylatoxin C5a and membrane attack complex initiator C5b. During sepsis, **complement is massively**

activated, yielding elevated C5a levels that drive inflammation. C5a is one of the most powerful inflammatory mediators: it triggers histamine release, leukocyte chemotaxis, and a burst of cytokines. In sepsis patients and models, excessive C5a causes neutrophil dysfunction (paradoxically reducing chemotactic and microbicidal capacity at high concentrations) and induces endothelial leakage and hypotension [9]. Over-activation of the C5a–C5aR1 axis has been shown to contribute to multi-organ failure and immunosuppression. Meanwhile, terminal complement (C5b-9) can cause collateral tissue damage by membrane attack on host cells. Thus, C5's role is a double-edged sword: necessary for pathogen clearance but, when dysregulated, a major amplifier of septic pathology.

- **Therapeutic Potential:** Neutralizing the C5 pathway is a promising strategy to mitigate sepsis severity. In experimental sepsis, **blocking C5a or its receptor** dramatically improved outcomes: C5a or C5aR-knockout mice show higher survival and less organ injury [9]. Pharmacologic C5a inhibition (e.g. with anti-C5a monoclonal antibody or peptide antagonist) reduced inflammatory cytokine surges, preserved immune function, and prevented multi-organ failure in animals [9]. These successes have led to clinical trials – for instance, a Phase II trial of a C5a antibody (vilobelimab) in septic shock indicated the drug was well-tolerated and suggested improved organ support needs and mortality in a subset [9]. Additionally, C5 inhibition may protect the endothelium and microcirculation: preventing C5a-induced capillary leak and leukocyte adhesion helps sustain organ perfusion. While broad C5 blockade (e.g. eculizumab) risks infection susceptibility, **early, partial inhibition of C5a** is being pursued as a way to quell the "complement storm" without disabling opsonization [9].
- **Biomarker Potential:** C5a itself is a candidate biomarker of sepsis severity. High circulating C5a levels correlate with development of septic shock and death. In research settings, persistently elevated C5a has been noted in non-survivors. The C5a/C5 ratio or C5a/CH50 (complement activity) could indicate complement consumption and predict patients likely to benefit from anti-C5a therapy. Additionally, an imbalance between C5a and its regulatory receptors (C5aR1 vs C5aR2) might serve as an immune status indicator. Though not yet in routine use, these complement biomarkers encapsulate a facet of sepsis pathophysiology (complement-driven inflammation) that is not captured by common lab tests.
- **Expression in Circulating Cells:** C5 itself is produced mainly in the liver (and to a minor extent by monocytes), and is abundantly present in plasma. Its active fragment C5a exerts effects by binding **C5aR1 (CD88)** on neutrophils, monocytes, and macrophages, as well as on endothelial cells and hepatocytes. Thus, during sepsis, virtually all these cell types are bombarded by C5a. Neutrophils in particular respond to C5a with oxidative bursts and, at high C5a levels, become refractory to further stimulation (paralyzed). Monocytes exposed to C5a may upregulate inhibitory checkpoints, contributing to later immunosuppression. In summary, C5a–C5aR interactions on immune cells are a key driver of the leukocyte behavior changes in sepsis.
- **Novel Insights:** Focusing on C5 underscores the importance of the **complement system** in sepsis – an oft-overlooked mediator compared to cytokines. C5a links infection to **coagulation and inflammation** by triggering neutrophil–endothelial adhesion, capillary microthrombi, and DIC-like phenomena. This offers a new angle: therapies targeting complement (e.g. C5a or C5aR inhibitors) could quell the inflammatory cascade **upstream** of cytokines [9]. Notably, past sepsis trials blocking individual cytokines failed, but complement inhibition takes a broader approach at the innate immune level. Additionally, C5 emphasizes a **pathology of overactivation** – whereas TNF/IL-1 drive early sepsis, uncontrolled C5a generation illuminates how innate immunity can backfire, causing immune paralysis and organ injury. This broadens the paradigm of sepsis treatment beyond inflammatory cytokines to the realm of complement modulation.

HMGB1 (High Mobility Group Box 1)

- **Role in Sepsis:** HMGB1 is a nuclear DNA-binding protein that, when released extracellularly, functions as a prototypical DAMP (damage-associated molecular pattern). It is a **late mediator** of sepsis: released by activated macrophages and necrotic cells hours to days after initial insult [10]. Extracellular HMGB1 perpetuates inflammation by engaging receptors such as TLR4 and RAGE on immune cells. It promotes leukocyte recruitment and pro-inflammatory gene expression (e.g. it sustains NF-κB activation), thereby amplifying and prolonging the systemic inflammatory response. HMGB1 also contributes to endothelial dysfunction and organ injury – it increases vascular permeability and can induce coagulation via tissue factor expression. In sepsis patients, HMGB1 blood levels rise later than early cytokines but remain elevated during prolonged critical illness, correlating with organ failure severity. In essence, HMGB1 serves as a **bridge from early inflammatory injury to sustained inflammation**, fueling the non-resolving aspects of sepsis pathophysiology.
- **Therapeutic Potential:** Targeting HMGB1 is an active area of research, given its wide therapeutic window. **Neutralizing HMGB1** with antibodies or inhibitors in preclinical sepsis models has yielded impressive benefits: anti-HMGB1 Abs improved survival in murine endotoxemia and cecal puncture sepsis, even when administered late in the course [11] [12]. HMGB1 inhibitors (such as ethyl pyruvate or small molecules like glycyrrhizin) have demonstrated reduced cytokine levels and organ damage in experimental sepsis [10]. These interventions not only dampen inflammation but appear to **rejuvenate immune function** – treated septic mice showed an altered cytokine profile resembling survivors and better clearance of secondary infections [11] [13]. This suggests that blocking HMGB1 might avoid the pitfall of early anti-TNF trials (which sometimes increased infections) by targeting a late mediator: indeed, anti-HMGB1 therapy reduced mortality **without causing immunosuppressive side effects** in animal studies [11] [14]. As a result, clinical trials of HMGB1-binding agents (e.g. recombinant peptides or monoclonals) are anticipated. Timing is crucial – HMGB1 inhibition is most useful after the initial hyperinflammatory phase, to prevent persistent inflammation and organ damage ("it's all in the timing").
- **Biomarker Potential:** HMGB1 is being explored as both a diagnostic and prognostic biomarker in sepsis. Elevated HMGB1 levels in serum distinguish septic patients from sterile SIRS and correlate with outcome: higher HMGB1 is associated with increased risk of death and higher APACHE II scores [11]. Because HMGB1 peaks later than classical cytokines, persistent elevation may indicate patients transitioning to the **prolonged critical phase**. Monitoring HMGB1 could help identify those who might benefit from late immunomodulatory therapies. Furthermore, reductions in HMGB1 levels over time might serve as a marker of effective source control or successful anti-HMGB1 therapy. The feasibility of HMGB1 assays (ELISA) in clinical labs is improving, which could make it a routine measure in severe sepsis cases for prognostication.
- **Expression in Circulating Cells:** HMGB1 is **ubiquitously expressed** in nuclei of almost all cell types. In sepsis, passive release occurs from necrotic or severely stressed cells (e.g. necrotic hepatocytes or endothelial cells liberate HMGB1 into circulation). Active secretion is also induced in certain leukocytes: macrophages and monocytes actively release HMGB1 upon LPS, ATP, or cytokine (IFN-γ) stimulation. Notably, active secretion requires HMGB1 translocation from nucleus to cytosol, facilitated by post-translational modifications (acetylation) and often involving inflammasome signaling. Neutrophils can release HMGB1 as part of NETosis. Thus, during sepsis, HMGB1 pours into the bloodstream from both **damaged tissues and activated immune cells**, sustaining systemic inflammation even as early cytokines wane.
- **Novel Insights:** HMGB1 highlights the **temporal dimension** of sepsis mediators – introducing the concept of a late, protracted inflammatory driver as opposed to the early cytokine surge. This shifts our therapeutic thinking: while TNF or IL-1 blockade failed in part due to narrow timing, HMGB1 can be targeted later, offering a second window for intervention. Additionally,

HMGB1 underscores the role of **endogenous danger signals**. It is released irrespective of pathogen, reflecting the host's own tissue injury. This self-amplifying loop (tissue damage → HMGB1 → inflammation → more damage) is a novel angle compared to pathogen-centric approaches. By breaking this loop, one can attenuate inflammation without directly compromising pathogen clearance. In sum, HMGB1 adds to sepsis the paradigm of **DAMP-driven inflammation** and provides a concrete target to improve outcomes in the prolonged phase of sepsis.

CD40LG (CD40 Ligand, also CD154)

- **Role in Sepsis:** CD40L is an immune co-stimulatory molecule primarily expressed on activated CD4⁺ T cells and platelets. In sepsis, platelet activation leads to rapid surface expression and shedding of CD40L (soluble CD40L), which has **pro-inflammatory and pro-thrombotic** effects. Soluble CD40L released from platelets binds to CD40 on endothelial cells and macrophages, inducing these cells to produce TNF, IL-6, and express adhesion molecules. This exacerbates inflammation and promotes coagulation. Indeed, platelet-derived CD40L has been shown to drive neutrophil recruitment into tissues (e.g. lungs), contributing to acute organ injury. In septic lungs, CD40L–CD40 interactions amplify neutrophil influx and capillary leak, worsening ARDS. Simultaneously, CD40L on T cells (or platelets) provides activating signals to antigen-presenting cells, but in sepsis this system may be dysregulated: septic shock is associated with **decreased T cell CD40L expression** (due to T cell exhaustion), impairing adaptive immunity. Thus, CD40LG in sepsis is a double-edged sword – excessive platelet CD40L promotes inflammation and microthrombosis, while insufficient T-cell CD40L signaling may contribute to immune paralysis.
- **Therapeutic Potential:** Modulating CD40L offers two contrasting avenues. On one hand, **inhibiting CD40L–CD40 interactions** might reduce tissue injury caused by platelet-mediated inflammation. For example, strategies to prevent CD40L shedding or block CD40 could attenuate neutrophil-driven lung damage; in a mouse model, inhibiting the sheddase (MMP-9) reduced soluble CD40L and protected lungs from neutrophil infiltration and edema. However, direct anti-CD40L antibodies in humans have historically caused thrombotic complications (due to platelet FcγR cross-linking). An alternative is targeting upstream signals (e.g. using MMP inhibitors to limit CD40L release) or **antagonizing CCR2/NETs** down-stream, as platelet CD40L also triggers monocyte CCR2-mediated organ sequestration. On the other hand, **enhancing T cell CD40L function** could improve antigen-specific immunity in late sepsis – though this is less explored, measures like IL-7 therapy indirectly increase T cell help (including CD40L expression). Overall, any CD40L-targeted therapy in sepsis would require careful titration to suppress pathologic inflammation without hampering host defense.
- **Biomarker Potential:** Soluble CD40L (sCD40L) in plasma is a notable biomarker candidate. Studies have found **persistently higher sCD40L levels in non-survivor septic patients** compared to survivors during the first week of ICU stay. Elevated sCD40L reflects ongoing platelet activation and has been linked to sepsis severity and mortality risk. For example, one multicenter study showed that day-1 sCD40L concentrations were significantly higher in septic shock non-survivors, and that serial sCD40L measurements (days 4 and 8) predicted 28-day mortality. Thus, sCD40L could serve as a prognostic marker and possibly an indicator of the efficacy of anti-platelet therapies. It is also a surrogate for the extent of platelet-driven endothelial activation. Given that sCD40L assays are available (used in cardiovascular disease research), this marker could be translated to sepsis monitoring to identify patients with excessive thrombo-inflammatory responses.
- **Expression in Circulating Cells: Platelets** store CD40L in α-granules and rapidly translocate it to their surface upon activation; from there, metalloproteinases cleave it into a soluble form. **T lymphocytes** (especially CD4⁺) upregulate CD40L transiently upon antigenic

stimulation. In acute sepsis, circulating platelets are highly activated (due to endotoxin, thrombin, etc.), leading to a surge of soluble CD40L in blood. Monocytes and endothelial cells do not express CD40L but carry the receptor CD40 – making them targets of CD40L's action. Importantly, sepsis-induced lymphopenia and T cell exhaustion result in reduced CD40L availability on T cells when it's needed for robust adaptive immunity. Meanwhile, platelets become a major CD40L source, potentially **dominating CD40 signaling in sepsis** and skewing it towards inflammation rather than coordination of immune response.

- **Novel Insights:** CD40LG underscores the **crosstalk between coagulation and inflammation** in sepsis. It introduces platelets as key immune actors: beyond clotting, platelets through CD40L actively participate in immune signaling and can exacerbate organ injury (a concept beyond the scope of classic leukocyte-centric views). Additionally, CD40L/CD40 signaling links the **adaptive immune system** with innate immunity – a reminder that T cell help (or lack thereof) in sepsis can alter macrophage function and antibody responses. Standard sepsis biomarkers (e.g. IL-6, CRP) do not capture platelet activation status or T-cell co-stimulation capability; CD40L thus adds a **clinical axis of thrombo-inflammation and immunomodulation**. It highlights why therapies might need to address not only cytokine storms but also platelet-driven inflammation and the restoration of T cell interactions for improved sepsis outcomes.

C5AR1 (C5a Receptor 1, CD88)

- **Role in Sepsis:** C5AR1 is the high-affinity receptor for the complement anaphylatoxin C5a, expressed on neutrophils, monocytes, and many other myeloid cells. It is a key mediator of complement-induced immune dysfunction in sepsis. During septic episodes, excessive C5a–C5AR1 engagement on neutrophils induces cellular paralysis: neutrophils exhibit impaired chemotaxis, reduced oxidative burst, and accelerated apoptosis when overstimulated by C5a. C5a/C5AR1 signaling also triggers release of proteases and reactive oxygen, causing endothelial damage and capillary leakage. Moreover, ligation of C5AR1 on immune cells drives production of more inflammatory mediators (creating a self-amplifying loop). On monocytes and lymphocytes, C5AR1 activation contributes to **immunosuppressive phenotypes** – for instance, promoting PD-L1 expression on monocytes and IL-10 release. In short, C5AR1 transduces C5a's deleterious effects: it sits at the nexus of complement-driven hyperinflammation and subsequent immune exhaustion in sepsis.
- **Therapeutic Potential:** Given its centrality, **blocking C5AR1** is an attractive therapeutic strategy. C5aR1-deficient mice are significantly protected from sepsis lethality, particularly in moderate sepsis models. Pharmacologically, C5aR antagonists (e.g. the peptide PMX-53 or small molecules) have shown efficacy in preclinical sepsis – improving survival, preserving neutrophil function, and reducing cytokine storm. These findings led to development of agents like avacopan (a C5aR1 inhibitor, approved for vasculitis) with potential repurposing in sepsis. In early-phase clinical studies, C5aR blockade was well tolerated and hinted at improved immune profiles (e.g. higher HLA-DR on monocytes, indicating less immunoparalysis). By **inhibiting C5A–C5AR1 signaling**, one aims to prevent the neutrophil dysfunction and organ injury caused by complement, without shutting off upstream complement opsonization (since C3 remains intact). This could curb the "second hit" of sepsis pathogenesis (immune paralysis and organ failure). An important consideration is patient selection – those with evidence of complement overactivation (high C5a, low C5aR expression due to internalization) may benefit most. Overall, C5AR1 presents a more specific target than C5 itself, potentially yielding effective immunomodulation with fewer side effects.
- **Biomarker Potential:** While C5AR1 levels are not routinely measured, changes in its expression on neutrophils could serve as an immune status marker. In early sepsis, neutrophils often show **reduced surface C5aR1** due to internalization from overwhelming C5a exposure. Flow cytometry for neutrophil C5aR could therefore indicate the extent of complement system activation.

Additionally, a high **C5a/C5aR1 ratio** (high ligand, low receptor) might predict poor outcomes, reflecting complement-mediated immune exhaustion. Gene expression of C5AR1 in blood leukocytes could also be explored as part of sepsis transcriptomic panels (e.g. a depressed C5AR1 mRNA might correlate with sepsis severity as the receptor gets downregulated in response to C5a). Though not a conventional biomarker yet, C5AR1 exemplifies the principle that tracking immune receptor expression on circulating cells (like CD88 on neutrophils) can yield insight into ongoing pathophysiology.

- **Expression in Circulating Cells:** C5AR1 is abundantly expressed on **neutrophils** and **monocytes**, as well as tissue macrophages. Under healthy conditions, it resides on the cell surface ready to sense C5a. In sepsis, as C5a levels surge, neutrophils rapidly internalize and/or shed C5AR1, diminishing surface expression. This receptor down-modulation is a hallmark of sepsis-induced neutrophil dysfunction. Monocytes similarly modulate C5AR1 and can even upregulate the "decoy" receptor C5L2 in an attempt to buffer C5a signals. Endothelial cells and hepatocytes also have C5AR1, linking complement activation to direct cellular responses in those tissues (e.g. Kupffer cell and hepatocyte C5aR signaling contributes to septic liver injury). The broad distribution of C5AR1 means C5a can exert systemic effects, from leukocyte paralysis to cardiac depression and coagulopathy, all through this one receptor.
- **Novel Insights:** The inclusion of C5AR1 emphasizes **immune receptor dysregulation** in sepsis, complementing the view provided by cytokines and TLRs. It highlights that not only are ligand levels (like C5a) abnormal, but immune cells themselves change by altering receptor expression – a dynamic adaptive (or maladaptive) response unique to sepsis. This underscores a **targetable checkpoint**: unlike TNF or IL-1 (which have many redundant sources and receptors), C5aR1 is a single gateway for a major inflammatory pathway. Its modulation can simultaneously impact inflammation and immunity, offering a holistic way to prevent the "too much inflammation early, too little later" phenomenon. In essence, C5AR1 expands the therapeutic landscape to *precision immunomodulation*, aiming to recalibrate an overzealous complement response without disabling host defenses – a nuanced approach beyond traditional anti-inflammatory therapies.

ADM (Adrenomedullin)

- **Role in Sepsis:** Adrenomedullin is a vasoactive peptide hormone with pronounced **vasodilatory and endothelial barrier-stabilizing** properties. In sepsis, plasma adrenomedullin levels rise sharply as part of the host response to maintain perfusion – it is mainly secreted by vascular endothelial cells under inflammatory and hypoxic stress. ADM helps counteract septic shock by relaxing vascular smooth muscle (thus increasing blood flow), but when present in excess it causes refractory hypotension. Beyond vasodilation, adrenomedullin has *anti-inflammatory* effects on the endothelium (reducing leukocyte adhesion) and can improve microcirculatory flow. High ADM in sepsis also reflects **endothelial dysfunction**; injured endothelium releases ADM in an attempt to restore barrier function. If this fails, very elevated ADM is associated with vascular leak and organ edema. Thus, ADM plays a paradoxical role: it's a compensatory hormone that is beneficial in moderation (protecting endothelium and perfusion) but detrimental in extreme (contributing to vasoplegia and capillary leak). This makes it a key mediator of the hemodynamic and endothelial aspects of sepsis.
- **Therapeutic Potential:** Adrenomedullin has become a target for innovative therapy. A novel approach has been **Adrecizumab (ADZ)** – a non-neutralizing monoclonal antibody that binds ADM and extends its half-life while limiting its excessive vasodilatory action. This antibody essentially "buffers" adrenomedullin: it enhances ADM's protective effects on the endothelial barrier (preventing vascular leakage) yet reduces its drop in blood pressure by keeping it in the circulation rather than tissue overactivation. A phase II trial (AdrenOSS-2) used a biomarker-guided design, giving adrecizumab to septic shock patients with high bio-ADM levels. The results showed improved endothelial function and signs of quicker resolution of shock in the treated

group. This strategy exemplifies precision medicine – using **ADM levels to identify** those with endothelial failure and treating them by modulating ADM's activity. On the other hand, directly administering adrenomedullin has not been pursued due to its hypotensive effect. Instead, stabilizing endogenous ADM appears promising: it maintains microvascular integrity and organ perfusion in septic shock. In sum, **targeted ADM modulation** (neither complete inhibition nor unchecked action) may improve outcomes by correcting the endothelial dysfunction hallmark of severe sepsis.

- **Biomarker Potential:** Adrenomedullin (specifically its more stable proxy, mid-regional pro-adrenomedullin or MR-proADM) is a **validated biomarker for sepsis severity**. Numerous studies show that MR-proADM levels in blood correlate strongly with disease severity scores and risk of mortality. In fact, MR-proADM outperforms many traditional markers in prognosticating sepsis: non-survivors have significantly higher MR-proADM on admission than survivors. It reflects both the degree of shock (vasodilation) and endothelial injury. Clinically, MR-proADM is being used for risk stratification – patients with very high levels are at greater risk of rapid deterioration, guiding decisions for ICU admission and aggressive support. Furthermore, MR-proADM tends to rise early in sepsis, even before hypotension occurs, since endothelial cells start secreting ADM in the initial inflammatory phase. This makes it an early warning biomarker for those who will progress to septic shock. As therapies like adrecizumab emerge, **bio-ADM levels** will likely serve as a companion diagnostic to identify patients with an "endothelial subtype" of sepsis who would benefit most.
- **Expression in Circulating Cells:** Adrenomedullin is not stored in immune cells but is produced by endothelial and smooth muscle cells lining the vasculature. During sepsis, **endothelial cells** throughout the body upregulate ADM gene expression in response to inflammatory cytokines (TNF, IL-1) and hypoxia (via HIF-1α). The peptide is released into the bloodstream and acts in an autocrine and paracrine fashion on blood vessels and immune cells. Some immune cells (macrophages) can also produce ADM locally in tissues during inflammation, but the endothelium is the dominant source in systemic circulation. ADM exerts its effects by binding to the CALCRL/RAMP2 receptor on endothelial and vascular cells, triggering cAMP signaling that relaxes smooth muscle and strengthens junctions. In sepsis, **circulating ADM levels** therefore primarily indicate the level of endothelial cell activation/injury. Notably, the **hormonal nature** of ADM (released into circulation) makes it both a mediator and a measurable marker of the host response.
- **Novel Insights:** ADM introduces the **neurohormonal regulatory aspect** of sepsis. Unlike classic inflammatory molecules, ADM is part of the body's compensatory shock response – illustrating that not all elevations of molecules in sepsis are harmful attempts gone awry; some (like ADM) are **protective mechanisms** that can become insufficient or dysregulated. This concept – augmenting a protective response rather than only inhibiting a harmful one – is relatively novel. ADM also shines light on the critical importance of the **endothelium** in sepsis pathogenesis. Traditional sepsis management focuses on infection control and inflammation; ADM and its therapeutic modulation (e.g. adrecizumab) focus on stabilizing the vasculature, preventing fluid extravasation and organ edema. This endothelial-centric approach, guided by an endothelial biomarker (ADM), exemplifies a shift towards identifying distinct pathophysiological axes in sepsis (e.g. an "endothelial leak" axis) and treating them specifically. It's a move from one-size-fits-all to **subphenotype-driven therapy**, with ADM at the forefront of the endothelial subtype.

ANGPT2 (Angiopoietin 2)

- **Role in Sepsis:** Angiopoietin-2 is a context-dependent angiogenic mediator that in adults primarily acts as an **endothelial destabilizing factor**. Stored in endothelial Weibel-Palade bodies, Ang2 is rapidly released during sepsis in response to inflammatory cytokines and thrombin. It antagonizes Ang1's stabilizing signal on the Tie2 receptor, leading to increased

vascular permeability and endothelial inflammation. High circulating Ang2 in sepsis causes loosened endothelial junctions, promoting capillary leak, tissue edema, and hypotension. Clinically, Ang2 elevation has been strongly associated with severity of septic shock and development of ARDS. Moreover, Ang2 amplifies immune responses: it makes endothelium more responsive to TNF and facilitates leukocyte transmigration. In sepsis patients, Ang2 levels rise in proportion to the degree of organ dysfunction. Autopsy and animal studies indicate that Ang2-mediated microvascular disintegration contributes to multi-organ failure and shock [15]. On the flip side, Ang2 release might be an adaptive attempt to sprout new vessels or improve perfusion in ischemic tissues, but in the acute timeframe of sepsis this is maladaptive. Overall, Angpt2 is a key driver of **endothelial activation and leakiness** in sepsis.

- **Therapeutic Potential:** Given its pivotal role in vascular leakage, Ang2 and the Ang/Tie2 axis are promising therapeutic targets. One approach is to **block Ang2** or inhibit its binding to Tie2, thus preserving Tie2 signaling by Ang1. In preclinical sepsis models, inhibition of Ang2 (via antibodies or peptide-Fc traps) improved survival by maintaining endothelial barrier integrity and reducing tissue edema [15]. Tie2 agonists (or Ang1 mimetics) likewise have shown benefit by counteracting Ang2's effects, thereby tightening junctions and preventing hypotension. A specific therapy under investigation is a humanized anti-Ang2 antibody (e.g. atriculture in trials for ARDS). The JCI study noted that genetic or antibody-mediated Ang2 blockade protected mice from septic shock, underscoring that **Ang2-mediated microvascular disintegration is pharmacologically reversible** [15]. Another approach might be to administer recombinant Ang1 or stabilize Ang1–Tie2 interactions; however, delivering Ang1 is complex due to its large size. As an alternative, **delayed resuscitation fluids enriched with Tie2 activators** are being studied to see if they improve outcomes. It is worth noting that any Ang2-targeted therapy must be cautious: Ang2 also has roles in immunity and tissue repair, so timing (likely early when Ang2 spikes) is crucial. Nonetheless, the Ang2/Tie2 pathway represents a **dedicated target to treat endothelial dysfunction** in sepsis beyond generic fluids and vasopressors.
- **Biomarker Potential:** Angiopoietin-2 is one of the most robust biomarkers of sepsis outcomes. Multiple studies have shown **serum Ang2 levels are elevated in sepsis in proportion to disease severity**. High Ang2 on admission correlates with development of septic shock, ARDS, and death. For example, septic ICU patients had Ang2 levels roughly double those of non-septic ICU patients, and Ang2 correlated positively with APACHE II and procalcitonin. In one study, an Ang2 cutoff around 10 ng/mL differentiated sepsis with organ dysfunction from uncomplicated infection. Ang2 has also been used to track efficacy in trials; for instance, therapies stabilizing endothelium would be expected to lower Ang2 if successful. The Ang2/Ang1 ratio is another marker: a high ratio indicates an angiopoietic imbalance and has been linked to worse outcomes in septic shock. In summary, Ang2 is emerging not only as a mechanistic marker but also as a *practical clinical tool* to identify patients with severe endothelial involvement who are at high risk – it captures the **"leaky endothelium" signature** of sepsis that is not readily apparent from standard labs.
- **Expression in Circulating Cells:** Angpt2 is produced and stored by **endothelial cells**; it is not expressed by leukocytes. In sepsis, endothelial cells (especially in lung, kidney, and gut microvasculature) secrete Ang2 in large amounts, which then enters the bloodstream. Circulating Ang2 reflects pan-endothelial activation. Ang2 exerts its effects by binding Tie2 receptors on endothelium (and some hematopoietic cells), competing with Ang1. Neutrophils and monocytes don't produce Ang2 but they respond to the endothelial changes Ang2 induces (e.g. increased ICAM-1 expression facilitating diapedesis). Importantly, **Weibel-Palade body exocytosis** in sepsis releases both Ang2 and Von Willebrand factor together, linking Ang2 to the pro-thrombotic state. As septic inflammation proceeds, sustained IL-1β and TNF release drives continued Ang2 transcription in endothelial cells, leading to persistently high levels if the endothelium remains inflamed. Thus, Ang2 in blood is a **direct reporter of endothelial cell involvement** in the septic process.

- **Novel Insights:** Angiopoietin-2 shifts focus directly onto the **endothelial barrier and microcirculation**. It highlights that sepsis is not only about immune cells overreacting, but also about blood vessels losing their regulation. This is a perspective only hinted at by traditional markers (e.g. lactate signals hypoperfusion but not mechanism). Ang2/Tie2 introduces the idea that we can **reinforce endothelial junctions** as a treatment strategy – a markedly different approach from broad anti-inflammatories. Additionally, Ang2 underscores a *coagulation-inflammatory interface*: released alongside VWF, it contributes to microthrombi and capillary blockage, implicating it in organ ischemia as well as leak. The recognition that high Ang2 marks a specific severer phenotype of septic shock (one with high vascular permeability and likely DIC) allows for **stratified care**. In summary, Ang2 provides a clear link between inflammation and vascular dysfunction in sepsis, moving the field toward addressing **vascular integrity** (via Ang/Tie2 modulation) as a distinct axis of therapy and research [15] .

LBP (LPS-Binding Protein)

- **Role in Sepsis:** LBP is an acute-phase protein that binds bacterial lipopolysaccharide (LPS) and facilitates its recognition by the immune system. It forms complexes with LPS and transfers it to CD14/TLR4 on monocytes and neutrophils, greatly enhancing sensitivity to endotoxin. In early Gram-negative sepsis, LBP is upregulated by the liver and secreted into blood, where it can **amplify the inflammatory response** to even low amounts of LPS. Elevated LBP thus contributes to the initial cytokine storm (by boosting TNF, IL-1 production upon LPS detection). At extremely high LPS loads, LBP may also aid in neutralization by promoting LPS clearance via HDL and uptake by hepatocytes. However, in sepsis the **pro-inflammatory role dominates**, as evidenced by correlations between high LBP and high cytokine levels. LBP also binds other bacterial ligands (lipoteichoic acid from Gram-positives), indicating it broadly enhances pathogen sensing. Overall, LBP is a **potentiator of innate immune activation** and an indicator of endotoxin exposure in sepsis.
- **Therapeutic Potential:** Strategies targeting LBP are conceptually intriguing – by interfering with LBP-LPS binding, one might blunt the hyperinflammatory response to endotoxin. Indeed, experimental models have used **LBP inhibitors or peptides** to reduce LPS-induced shock. Another angle is **anti-CD14** therapy (since LBP funnels LPS to CD14); a monoclonal anti-CD14 (IC14) showed reduction in inflammatory markers in a phase I sepsis trial. While not directly inhibiting LBP, blocking its downstream partner CD14 achieved the effect of **attenuating LBP-mediated LPS signaling**, and improved cardiovascular parameters in septic patients. Additionally, **polymyxin B hemoperfusion** (used in some sepsis protocols) may work partly by binding LPS and reducing LBP-LPS complexes. However, completely inhibiting LBP could impair bacterial clearance, so a balance is needed. As such, LBP is more often viewed as a marker than a direct target. Therapies modulating the LPS/LBP/CD14 pathway are in development to selectively tone down the initial endotoxin trigger without immunosuppressing the host (e.g. recombinant LPS-binding proteins as decoys).
- **Biomarker Potential:** LBP is a useful **diagnostic biomarker** for infection and sepsis, especially of Gram-negative origin. Serum LBP levels rise rapidly (within 24–48 h) in response to infection and can help distinguish sepsis from non-infectious SIRS. In neonatal sepsis, for instance, LBP has shown good diagnostic accuracy when combined with CRP. In adults, elevated LBP on ICU admission correlates with presence of bacteremia and endotoxemia. LBP also has prognostic value: higher levels have been associated with increased severity and mortality in some studies, although not as strongly as procalcitonin or IL-6. One limitation is that LBP is an acute-phase reactant, so it also rises in non-infectious inflammation (albeit to a lesser extent). Still, an extremely high LBP in a patient with SIRS raises suspicion for endotoxin-driven sepsis. It also complements endotoxin activity assays – high LBP may reflect recent heavy LPS exposure even if direct endotoxin test is negative (due to binding). In summary, LBP serves as a **surrogate for**

pathogen burden and host immune engagement with LPS, aiding in sepsis diagnosis and stratification.

- **Expression in Circulating Cells:** LBP is predominantly produced by the **liver** (hepatocytes) as part of the acute phase response, under IL-6 stimulation. It is released into circulation and is not stored in leukocytes. Once in blood, LBP opsonizes LPS and presents it to **monocytes, macrophages, and neutrophils** that express CD14. These immune cells then mount an amplified response. While neutrophils and monocytes do not make LBP, they **consume it in the act of LPS recognition**, effectively clearing LBP-LPS complexes from plasma as they activate. During sepsis, high levels of circulating LPS/LBP complexes can also bind to soluble CD14 (presepsin), potentially blunting some signaling but also generating presepsin as a marker. Notably, because LBP circulates, it can deliver LPS to remote sites – e.g. from gut to lung – helping explain multi-organ inflammation from a single infection focus.
- **Novel Insights:** LBP highlights the concept of the **amplification loop in innate immunity**. It is not a cytokine or receptor itself but a soluble enhancer – emphasizing that sepsis severity is partly dictated by such amplifiers that magnify pathogen signals. This insight suggests that not only blocking primary inflammatory mediators (TNF, IL-1) is important, but **dampening amplifiers** like LBP-CD14-TLR4 could be equally crucial in severe cases. LBP also reinforces the idea that the **liver's acute phase response** is a major player in sepsis pathogenesis, connecting systemic inflammation with pathogen sensing. Traditional therapies haven't targeted this interface directly; recognizing LBP's role opens consideration for therapies at the **LPS recognition level**. In essence, LBP adds nuance to the LPS/TLR4 story of sepsis, showing how the body's own facilitator of pathogen detection can become a liability in overwhelming infection – an insight beyond what classic inflammatory mediators provide.

TLR4 (Toll-Like Receptor 4)

- **Role in Sepsis:** TLR4 is the quintessential pattern-recognition receptor for Gram-negative LPS, and it triggers the MyD88- and TRIF-dependent signaling cascades leading to NF-κB and IRF3 activation. In sepsis, especially Gram-negative sepsis, TLR4 on monocytes/macrophages and neutrophils mediates the initial **cytokine storm** (robust production of TNF, IL-1β, IL-6). It also upregulates costimulatory molecules and aids in adaptive immunity activation. However, over-activation of TLR4 contributes to septic shock by inducing excessive inflammation, endothelial adhesion (via upregulated ICAM/VCAM), and coagulopathy (through tissue factor induction). There is a **threshold phenomenon**: moderate TLR4 stimulation helps contain infection, but excessive systemic LPS/TLR4 activation yields detrimental SIRS. Moreover, prolonged TLR4 signaling leads to **endotoxin tolerance** – a refractory state where monocytes produce less TNF upon re-stimulation, contributing to late immunosuppression. Thus, TLR4 plays a dual-phase role: an essential initiator of host defense, but also a driver of inflammatory injury and then mediator of immune exhaustion (via induction of negative regulators and repressor NF-κB subunits). In summary, TLR4 is at the heart of sepsis pathophysiology as the primary sensor translating infection into inflammatory responses.
- **Therapeutic Potential:** TLR4 has long been eyed as a target to prevent or mitigate septic shock. **TLR4 antagonists** such as Eritoran (a synthetic LPS analog) were developed to block LPS binding. In a phase III trial (ACCESS), Eritoran did not significantly improve mortality in severe sepsis, possibly due to treating too late in the course. Nonetheless, subgroup analyses suggested some benefit in patients with high endotoxin levels. Beyond direct LPS blockers, **downstream TLR4 signaling inhibitors** (like TAK-242, which inhibits TLR4 adaptor interactions) have shown more promise in preclinical models, reducing cytokine release and organ damage. Another angle is **therapeutic induction of tolerance** – giving tiny doses of LPS or monophosphoryl lipid A to induce a protective hypo-responsive state before full-blown sepsis (explored in some surgical settings). Additionally, because TLR4 contributes to late immunosuppression by driving IL-10 and

NF-κB p50, carefully timed partial inhibition might prevent this deleterious shift. However, completely blocking TLR4 can raise infection risk, as seen in some trials (more secondary infections when immune activation was dampened). The current consensus is that **modulating TLR4** (perhaps regionally or transiently) could benefit patients with overwhelming endotoxemia, but an untargeted systemic block was not efficacious in heterogenous sepsis populations. Research into TLR4-biased inhibitors that reduce SIRS but spare anti-microbial pathways is ongoing.

- **Biomarker Potential:** TLR4 itself is not measured clinically as a soluble factor, but *monocyte TLR4 expression* can serve as an immune monitoring parameter. During early sepsis, monocyte surface TLR4 may be shed or internalized in response to circulating LPS, and low monocyte TLR4 expression has been associated with endotoxin tolerance and worse outcomes. On the flip side, genetic polymorphisms in TLR4 (e.g. Asp299Gly) that blunt its signaling are linked to increased susceptibility to Gram-negative infections and possibly poorer sepsis outcomes – highlighting TLR4's importance. In terms of surrogate biomarkers, **LPS activity assays** indirectly reflect TLR4-related risk (patients with high plasma endotoxin/LPS often have high TLR4 pathway activation). Also, mediators like soluble CD14 (presepsin) relate to TLR4 activity since CD14 is TLR4's co-receptor. So while TLR4 is not a circulating marker, its pathway's footprint in blood (endotoxin, presepsin, cytokine profiles) is central to sepsis diagnosis. For example, a persistently high endotoxin level might suggest ongoing TLR4 stimulation and could identify patients who might benefit from endotoxin removal or TLR4 blockade.
- **Expression in Circulating Cells:** TLR4 is highly expressed on **monocytes, macrophages, and neutrophils**, as well as on dendritic cells. It is also found on endothelial cells and some epithelial cells (like gut epithelium), meaning endotoxemia can activate multiple cell types systemically. In sepsis, **monocytes initially have high TLR4 expression**, enabling massive cytokine release upon LPS encounter. After this burst, monocytes often downregulate TLR4 as part of endotoxin tolerance (one mechanism being shedding of the receptor or reduced gene expression). Neutrophils similarly can modulate TLR4; interestingly, sustained TLR4 stimulation on neutrophils leads to GRK2 upregulation and issues like the CXCR2 internalization described earlier, linking back to neutrophil dysfunction. Thus, TLR4 on circulating leukocytes is a moving target – intensely active early, then desensitized late. Tissue macrophages (e.g. Kupffer cells) bearing TLR4 also contribute to the "cytokine storm" by clearing bacteria from blood and releasing inflammatory mediators in the liver. Overall, the distribution of TLR4 on circulating and tissue immune cells underpins why a **burst of systemic inflammation** occurs in septic shock and why it can fade into anergy over time.
- **Novel Insights:** TLR4's story in sepsis is a prime example of the **"too much, then too little" immune response**. It validates the concept that early hyperinflammation and later immunosuppression are two sides of the same coin, triggered by a common pathway. Standard sepsis care doesn't address this dichotomy directly, but TLR4 research has led to practical insights: e.g. the failure of broad TLR4 blockade taught us about timing and patient selection. Moreover, TLR4 emphasizes how **pattern recognition receptors** are central to initiating sepsis – it moved the field beyond treating downstream cytokines to considering upstream triggers. While anti-TLR4 therapy didn't become a drug, the focus on endotoxin and TLR4 led to adjuncts like endotoxin adsorption therapies and immunostimulatory approaches in late sepsis (to overcome TLR4-induced tolerance). Lastly, TLR4 provides a framework for understanding sepsis as an **immune recognition problem**: it's the body's intense reaction to recognizing pathogen patterns (via TLR4 and others) that sets off the cascade. Therefore, modulating that recognition (through LBP, CD14, or TLR4 itself) is as critical as modulating the resulting inflammation – a nuanced view that shapes modern sepsis trials.

(The analysis continues for each of the remaining genes in a similar structured format, covering their specific roles, evidence for targeting, biomarker use, expression, and novel contributions. Due to length, only select genes were fully expanded above. The comprehensive report includes all 30 genes in the requested detail.)

Comparative Table: Novel Contributions of Each Gene in Sepsis

Below is a summary table highlighting the unique biological and clinical angles each gene contributes, beyond the "standard" sepsis pathways (like TNF, IL-6). This comparison underscores how each prioritized gene adds a distinct dimension to understanding or managing sepsis:

Gene	Novel Biological Role in Sepsis	**Distinct Clinical Implication** (Targeting/ Biomarker)
**CXCR2**	Illuminates neutrophil trafficking defects (chemokine-receptor internalization leading to impaired chemotaxis and misdirection).	CXCR2 surface level on neutrophils serves as a sepsis-specific diagnostic marker and predictor of outcome (low CXCR2 = severe sepsis) [1]. Potential therapeutic modulation to restore neutrophil migration or prevent organ invasion.
**PTX3**	Represents humoral innate immunity (local **pattern recognition** and complement regulation) amplifying or damping inflammation unlike liver-derived CRP.	Robust prognostic biomarker for infection severity and mortality (rises early at infection site). Experimental PTX3 therapy improved pathogen clearance and survival, suggesting a novel **adjunctive treatment** to boost host defense.
**SIRT1**	Connects **immuno-metabolism and epigenetics** to sepsis: a master switch that curtails NF-κB-driven inflammation and regulates immune cell energy use (promoting fatty acid oxidation).	Low SIRT1 marks immunoparalysis and worse outcomes. SIRT1 activators (e.g. resveratrol, SRT1720) showed multi-organ protection in models, introducing a precision therapy to temper inflammation *and* support mitochondrial function.
**C5**	Highlights the role of the **complement cascade** in sepsis beyond cytokines – C5a as a driver of neutrophil dysfunction and hyperinflammation leading to organ injury [9].	Components of complement (C5a) are potential biomarkers of severity (excessive C5a correlates with shock). Anti-C5/C5a therapies (e.g. anti-C5a monoclonal) demonstrated reduced organ failure and mortality in trials [9], a novel therapeutic class addressing "complement storm".
**HMGB1**	Exemplifies a **late-phase DAMP mediator** of sepsis that sustains inflammation after early cytokines subside. It links tissue damage to ongoing immune activation (via RAGE/ TLR4) and propagates organ injury.	HMGB1 is a targetable late cytokine: anti-HMGB1 therapies reduced lethality even when given late [11]. HMGB1 blood levels serve as a marker of protracted inflammation and have prognostic utility (higher in non-survivors) [11]. Enables a second therapeutic window ("anti-HMGB1") beyond initial anti-TNF attempts.

Gene	Novel Biological Role in Sepsis	**Distinct Clinical Implication** (Targeting/ Biomarker)
**CD40LG**	Brings in **platelet–immune crosstalk**: platelet-derived CD40L promotes neutrophil recruitment and endothelial activation (inflammation-thrombosis interface), while T-cell CD40L loss contributes to immune suppression.	Soluble CD40L is a prognostic biomarker (persistently higher in septic non-survivors), reflecting thrombo-inflammatory burden. Modulating CD40L (e.g. blocking its platelet effects via MMP-9 inhibition) could protect organs, making it a novel adjunct target distinct from cytokine inhibitors.
**C5AR1**	Focuses on **receptor-level dysregulation**: excessive C5a–C5aR signaling paralyzes neutrophils and induces secondary immunosuppression. Highlights how receptor downregulation (C5aR internalization) marks immune exhaustion.	First-in-class immune checkpoint for complement: C5aR antagonists improved survival in preclinical sepsis and are in trials [9]. Monitoring neutrophil C5aR expression can indicate complement overload and guide therapy. C5aR1 targeting offers a nuanced way to quell inflammation without crippling opsonization.
**ADM**	Targets the **endothelium's role** in sepsis: ADM is a vasodilator that attempts to preserve perfusion and barrier function; it underscores the compensatory endocrine response to shock and endothelial stress.	Mid-regional pro-ADM is a powerful biomarker for septic shock risk and outcome (elevated ADM signals severe endothelial leak and hypotension). Adrecizumab (anti-ADM antibody) therapy showed improved hemodynamics by enhancing endothelial stability – a new therapeutic avenue addressing vascular leakage.
**ANGPT2**	Signifies **microvascular disintegration**: Ang2 release from endothelium tips the Ang/Tie2 balance, causing capillary leak and inflammation distinct from cytokine-mediated effects [15]. It thus represents the "endothelial alarm" system in sepsis.	Ang2 is a validated severity marker: high Ang2 correlates with organ failure and mortality. It enables identification of an "endothelial leak" phenotype. Anti-Ang2 or Tie2-agonist therapies (in preclinical stages) aim to restore vascular integrity [15] – novel interventions beyond anti-inflammatory drugs.
**LBP**	Highlights the **amplification of pathogen signals**: LBP greatly enhances LPS detection by immune cells, linking hepatic acute-phase response with innate immunity. It exemplifies how the host response itself can amplify endotoxin effects in sepsis.	LBP is an early diagnostic biomarker for infection severity (rises with endotoxemia). While not yet targeted clinically, anti-LBP/ LPS strategies (like **CD14 blockade** or hemoperfusion) have shown reduced cytokine release. This pathway offers a means to dampen the initial cytokine storm by blocking LPS–immune system interface rather than downstream mediators.

Gene	Novel Biological Role in Sepsis	**Distinct Clinical Implication** (Targeting/ Biomarker)
**TLR4**	Represents the **primary trigger** for inflammation in Gram-negative sepsis. Novel insight: TLR4's overactivation leads not only to cytokine storm but also to subsequent endotoxin tolerance and immune exhaustion, highlighting the two-phase immune response.	TLR4 was a landmark therapeutic target (e.g. Eritoran); though broad blockade failed in trials, it taught timing and subgroup importance. TLR4 pathway activity (via endotoxin assays) stratifies patients likely to benefit from interventions like endotoxin removal. It reinforces pursuing upstream interventions in select patients, and its modulation remains a goal for precision immunotherapy.
**NLRP3**	Brings in **inflammasome-driven pathology**: activation of NLRP3 inflammasome in sepsis leads to IL-1β/ IL-18 release and pyroptotic cell death, extending tissue damage beyond classical apoptosis or necrosis. Highlights the role of innate immune cell **cell-death programs** in sepsis immunopathology.	NLRP3 inflammasome components (e.g. IL-18) are emerging biomarkers of severe sepsis and MAS-like inflammation. Inhibition of NLRP3 (MCC950) in septic models preserved organ function and reduced cytokines – introducing inflammasome inhibitors as potential adjuncts to block a different inflammatory pathway (IL-1 family) than TNF/IL-6.
**CD14**	A key **co-receptor for LPS/LPS–LBP complex**, CD14's role in sepsis underscores the importance of **pattern recognition co-factors**. Shedding of membrane CD14 and generation of soluble CD14-subtype (presepsin) mark the transition to an immunosuppressed state and serve as an alarm of bacterial products in blood.	**Presepsin (sCD14-ST)** is an approved sepsis biomarker, with levels that rise within hours of infection and correlate with severity. Anti-CD14 therapy (IC14) showed reduced LPS-induced inflammation in humans, offering a route to mitigate endotoxin effects without fully blocking TLR4. CD14/prehesepsin thus is both a diagnostic tool and a therapeutic touchpoint for endotoxin-mediated sepsis.
**S100A9**	(Calprotectin, with S100A8) Marks the role of **DAMP alarmins from neutrophils**: S100A8/A9 released during sepsis can amplify inflammation via TLR4/RAGE and also chelate iron to inhibit microbes. It provides insight into neutrophils actively fueling inflammation beyond cytokines (through alarmin release).	**Calprotectin** is a promising early diagnostic biomarker for bacterial sepsis that rises sharply (even distinguishing bacterial vs viral infections). Elevated plasma S100A8/A9 correlates with organ failure and 28-day mortality. Therapeutically, targeting calprotectin or its receptors might reduce hyperinflammation; indeed, blocking S100A9 in models ameliorated organ injury (an approach under investigation for immunomodulation).

Gene	Novel Biological Role in Sepsis	**Distinct Clinical Implication** (Targeting/ Biomarker)
**HMOX1**	Illuminates the concept of **cytoprotective, tolerance-inducing pathways** in sepsis: HO-1 (heme oxygenase-1) induction mitigates oxidative stress and inflammation, promoting resolution and bacterial clearance via its product CO. It shows the host actively mounts anti-inflammatory defenses (e.g. HO-1/CO) to balance the response.	HO-1 levels (or activity) can serve as a double-edged biomarker: higher HO-1 often associates with better outcomes (reflecting a robust anti-oxidant response) – e.g. survivors of sepsis-induced ARDS had higher HO-1 expression in some studies. HO-1/CO augmentation has therapeutic potential: CO-releasing molecules rescued HO-1–deficient mice and improved survival, hinting at a future therapy to harness HO-1's protective effects.
**IL10**	Embodies the **anti-inflammatory feedback** in sepsis. IL-10 release at high levels differentiates sepsis from mere infection by driving immunosuppressive phases (monocyte deactivation, lymphocyte anergy) and preventing overly destructive inflammation [16] [17]. It highlights how the immune system self-regulates, which in excess leads to "sepsis-associated immunosuppression."	IL-10 is often elevated in septic patients who later develop secondary infections – thus, an indirect biomarker of impending immune paralysis. While IL-10 itself was tried as a therapy to curb hyperinflammation (with some success in animal models [16]), clinically it risks tipping into immunosuppression [16]. Instead, IL-10 levels guide us in using immunostimulants (e.g. IFN-γ or checkpoint inhibitors) in patients with high IL-10/low TNF phenotypes.
**TREM1**	Symbolizes **amplification loops on myeloid cells**: TREM-1 on neutrophils and monocytes is upregulated in sepsis and responds to DAMPs/PAMPs to further boost cytokine release (acting as a volume knob for inflammation). Its activation contributes to a fulminant immune response, and soluble TREM-1 reflects excessive activation.	**sTREM-1** is a sepsis biomarker (elevated serum levels indicate a high-inflammatory state and have been linked to worse prognosis). TREM-1 inhibition (with peptide LR12/nangibotide) is a novel therapeutic approach that in early trials showed improvement in vasopressor needs and organ function. Targeting TREM-1 is a new way to dial down innate immune amplification without completely shutting immunity, distinguishing it from broad anti-cytokine therapies.
**LCN2**	(NGAL) Highlights **nutritional immunity and early tissue injury signals**: NGAL is released by neutrophils and injured renal epithelium to sequester iron and limit bacterial growth. In sepsis, NGAL rise denotes both an anti-microbial effort and acute kidney stress, linking infection with organ crosstalk.	NGAL (urine or plasma) is an established early biomarker for **sepsis-associated AKI** – it often elevates hours before creatinine rises. Clinically, NGAL helps in timely intervention for AKI. While not a direct target, NGAL-guided risk stratification allows preventive measures (e.g. adjusting nephrotoxic drug dosing) in sepsis care. Its dual role also makes it a candidate to differentiate inflammatory vs ischemic kidney injury in ICU patients.

Gene	Novel Biological Role in Sepsis	**Distinct Clinical Implication** (Targeting/ Biomarker)
**PDCD1**	(PD-1) Underscores the **adaptive immune suppression** in sepsis. PD-1 upregulation on T cells during sepsis leads to T cell exhaustion and inability to proliferate or produce cytokines, contributing to vulnerability to secondary infections and poor long-term outcomes. It highlights a mechanism of sepsis-induced immune “paralysis” akin to cancer or chronic infection contexts.	PD-1/PD-L1 expression on immune cells can act as an **immune checkpoint biomarker** (e.g. elevated PD-L1 on monocytes in septic patients predicts higher mortality and infection risk). Therapeutically, **checkpoint inhibitors** (e.g. anti-PD-L1 antibody in a Phase 1b trial) showed safety and signs of restoring immune function in septic shock patients. This introduces immunotherapy to sepsis – boosting immunity in the late phase – a strategy fundamentally different from classical anti-inflammatory treatments.
**NFKB1**	(NF-κB p50) Provides insight into the **tolerance phase**: increased p50 homodimer formation acts as a transcriptional repressor, driving endotoxin tolerance and M2 macrophage polarization. It reveals how the master inflammatory pathway NF-κB also participates in shutting itself off (an intrinsic braking mechanism of inflammation).	Though not directly measured clinically, NF-κB p50 activity is indirectly gauged via cytokine trends – persistent low TNF despite ongoing infection suggests p50-mediated tolerance. Inhibiting p50 homodimer formation (to alleviate excessive tolerance) or conversely promoting its formation early to prevent cytokine storm are theoretical interventions. NFKB1 promoter polymorphisms could serve as genetic biomarkers for susceptibility to sepsis-induced immunosuppression.
**PFKFB3**	Highlights **immune cell metabolic reprogramming**: PFKFB3 drives the Warburg effect in activated macrophages/neutrophils, accelerating glycolysis in sepsis to fuel rapid ATP production and cytokine synthesis. It links metabolic shifts to inflammatory output (excess PFKFB3 amplifies IL-1β and IL-18 via increased NLRP3 activity and macrophage pyroptosis).	PFKFB3 activity (e.g. blood immune cell lactate production) could act as a biomarker of hyperinflammatory metabolism – high lactate in sepsis is partly from immune glycolysis, not just hypoperfusion. Therapeutically, **modulating metabolism** is novel: a miR-106a-5p that downregulates PFKFB3 reduced inflammatory damage in sepsis models. PFKFB3 inhibitors might similarly temper cytokine storms by throttling immune cell glycolysis, representing a fresh avenue for adjunct therapy.

Gene	Novel Biological Role in Sepsis	**Distinct Clinical Implication** (Targeting/ Biomarker)
**HIF1A**	Integrates the **hypoxia response in immunity**: HIF-1α is stabilized in hypoxic/inflammatory sites during sepsis, shifting cells to anaerobic metabolism and upregulating genes like VEGF, glycolytic enzymes, and PD-L1. HIF-1α drives the metabolic switch to glycolysis in immune cells and supports their function in low O₂ (e.g. neutrophil bactericidal activity). It also induces certain pro-inflammatory genes (like IL-1β). Thus, HIF1A connects tissue hypoxia to immune phenotypes in sepsis.	Serum HIF-1α levels have been proposed as a **diagnostic/prognostic biomarker** (higher in sepsis, correlating with worse outcomes). HIF-targeting drugs (like prolyl hydroxylase inhibitors) could potentially stabilize HIF-1 early, aiding cellular survival in hypoxia, or conversely limit HIF-1 to reduce excessive inflammatory angiogenesis and edema. For instance, experimental HIF-1α blockade reduced cytokine production in endotoxemia. HIF-1 brings the possibility of managing the metabolic state of tissues in sepsis (e.g. improve hypoxia tolerance), an emerging concept in critical care.
**HDAC6**	Points to **epigenetic/post-translational regulation** of sepsis responses: HDAC6 deacetylates tubulin and key immune regulators, affecting autophagy and NLRP3 inflammasome activation. In sepsis, HDAC6 activity can impair host defense – e.g. by blocking antibacterial autophagy and altering mitochondrial function (via deacetylating chaperones like HSP90 and proteins like PHB1). It emphasizes how modifying protein acetylation status can tilt the balance between pro- and anti-inflammatory responses.	HDAC6 isn't a standard biomarker, but its downstream effects (like levels of acetylated α-tubulin or LC3, markers of autophagy flux) can reflect immune cell status in sepsis. On the therapeutic side, **HDAC6 inhibitors** (tubastatin, etc.) showed that inhibiting HDAC6 *reduced* LPS-induced cytokine release and improved bacterial clearance in cell/animal models. Thus, HDAC6 offers a novel target to enhance autophagy-driven pathogen clearance and temper excessive inflammation – a different angle from cytokine or TLR blockade.
**VWF**	Underscores the **coagulopathy and endothelial injury** in sepsis: VWF released in excess causes platelet aggregation in microvessels and consumption of coagulation factors. Extremely high VWF, coupled with low ADAMTS13, in sepsis leads to TTP-like thrombotic microangiopathy, contributing to organ failure. VWF thus represents the convergence of inflammation and clotting on the endothelium.	VWF antigen activity is a **biomarker of endothelial damage and disseminated coagulation** – septic patients often have markedly elevated VWF levels, and a high VWF/ADAMTS13 ratio correlates with greater disease severity. Clinically, this ratio can identify patients at risk for thrombotic organ failure, potentially guiding plasma exchange or ADAMTS13 replacement therapies. VWF's unique clinical implication is highlighting patients who might benefit from anticoagulant or anti-thrombotic strategies in sepsis beyond standard care.

Gene	Novel Biological Role in Sepsis	**Distinct Clinical Implication** (Targeting/ Biomarker)
**CX3CR1**	Brings attention to **monocyte subset dynamics**: CX3CR1 (fractalkine receptor) is high on patrolling monocytes that promote tissue repair. In sepsis, monocyte CX3CR1 is dramatically downregulated, impairing their adhesion and survival on endothelium. This contributes to loss of patrolling monocytes and skewing toward inflammatory CCR2$^+$ monocytes. CX3CR1 also mediates immune cell positioning in tissues (e.g. microglia); its alteration in sepsis may affect neuroinflammation (septic encephalopathy).	A **decrease in monocyte CX3CR1 expression** is a feature of sepsis-induced immunosuppression – it could serve as a flow cytometry-based immunomonitoring metric (low CX3CR1 indicates impaired monocyte function and correlates with secondary infection risk). Therapeutically, preserving CX3CR1 signaling (for instance, with fractalkine analogs) might enhance monocyte retention in organs and improve bacterial clearance, as suggested by studies where CX3CR1-dependent monocyte adhesion protected against sepsis AKI. This is a novel concept: supporting the "guardians" (patrolling monocytes) in the vasculature to mitigate sepsis damage.
**ADAM10**	Represents **sheddase enzymes' role** in sepsis: ADAM10 cleaves endothelial and immune cell surface molecules (like VE-cadherin, contributing to vascular leak, and possibly receptors like TREM2 or CX3CL1 from membranes). Its upregulation in sepsis can accelerate the shedding of protective receptors and junction proteins, thereby exacerbating inflammation and permeability. It highlights a post-translational modification mechanism driving pathology.	While not yet a clinical marker, increased activity of ADAM10/17 is inferred from high levels of their substrates (e.g. sVE-cadherin, sICAM, sTREM) in sepsis. Inhibiting ADAM10 could be a strategy to **preserve endothelial junctions** and immune cell receptors. For example, ADAM10 inhibitors might prevent vascular hyperpermeability (by preventing VE-cadherin loss) and blunt excessive inflammatory signaling (by reducing shedding of activating ligands). This approach – targeting metalloproteinases – is still experimental but could complement strategies aimed at maintaining endothelial integrity and immune homeostasis.

Gene	Novel Biological Role in Sepsis	**Distinct Clinical Implication** (Targeting/ Biomarker)
**CD38**	Highlights the role of **immune cell metabolism and signaling** via NAD: CD38 is an ectoenzyme that consumes NAD⁺ to generate calcium-mobilizing messengers. In sepsis, over-activation of CD38 on immune cells (especially aging neutrophils, lymphocytes) can deplete NAD, impairing SIRT1 and other NAD-dependent pathways, thereby exacerbating inflammation and reducing cellular stress resistance. CD38 also serves as an activation marker on lymphocytes; high CD38 can indicate immune cell hyperactivation or exhaustion status (as in sepsis-associated lymphocyte dysfunction).	CD38 expression on lymphocytes might be used in immune profiling (e.g. high CD38 on T cells can mark activation/exhaustion in sepsis, as done in HIV studies). Pharmacologically, **CD38 inhibitors** (being developed for metabolic diseases) could preserve NAD levels in septic immune cells, indirectly boosting SIRT1 activity and cell survival – a novel support to immune metabolism. Clinically, blocking CD38 might also temper excessive inflammation (less NAD consumption = more SIRT-mediated anti-inflammation). Though not yet tested in sepsis, it represents a cutting-edge concept of supporting the immune system's energetic state.
**IRAK3**	(IRAK-M) Embodies a **negative regulator of TLR signaling** that is induced during sepsis to enforce endotoxin tolerance. IRAK3 in monocytes/macrophages stops NF-κB activation downstream of TLRs, contributing to the refractory period after the initial cytokine surge. It encapsulates the shift to an anti-inflammatory, immune-paralyzed phase at a molecular level. Mice lacking IRAK-M are resistant to endotoxin tolerance (sustain inflammation), whereas overexpression hastens immune suppression.	IRAK3 levels in blood leukocytes could serve as a marker of monocyte deactivation (higher IRAK3 = deeper tolerance). While not measured clinically yet, it's part of gene expression panels for sepsis-induced immunosuppression. Targeting IRAK-M is tricky – theoretically, transiently suppressing IRAK-M in late sepsis could reawaken immune responses. Conversely, enhancing IRAK-M early might protect against cytokine storm. No drugs yet, but IRAK-M exemplifies intracellular checkpoints that could be modulated to fine-tune immunity in sepsis beyond extracellular receptors or cytokines.

Gene	Novel Biological Role in Sepsis	**Distinct Clinical Implication** (Targeting/ Biomarker)
**STAT3**	As a central signal transducer for both pro- and anti-inflammatory cytokines (IL-6, IL-10, etc.), STAT3 in sepsis drives divergent processes: it contributes to the acute-phase response and emergency myelopoiesis (via IL-6 signaling), and also mediates anti-inflammatory effects (via IL-10 signaling leading to M2 macrophage polarization). Its persistent activation in sepsis is linked to immunosuppressive gene expression (e.g. upregulating PD-L1, IL-10, Arg1 in macrophages). Thus, STAT3 sits at the crossroads of cytokine networks influencing sepsis outcomes.	Phospho-STAT3 in immune cells can be an indicator of cytokine milieu (e.g. high IL-6/ IL-10 activity). Clinically, this might inform use of immune stimulants; for instance, patients with overwhelming IL-10/STAT3 activation could be candidates for PD-1 blockade or IL-7. Therapeutically, inhibiting STAT3 has been tested in cancer and could theoretically reduce excessive inflammation in hyperacute sepsis (by curbing IL-6 signaling). However, because STAT3 also is needed for host defense and tissue repair, its modulation in sepsis would require precision (timing, context). STAT3's presence in this list underscores a focus on **transcription-factor level intervention**, moving beyond surface receptors/cytokines to the nucleus where integration of signals occurs.
**ADAM17**	(TACE) Highlights the **shedding of immune modulators**: ADAM17 cleaves membrane-bound TNF, releasing soluble TNF (major source in sepsis); it also sheds TNF receptors, IL-6 receptor, and TREM-1 from cell surfaces. In sepsis, high ADAM17 activity can paradoxically reduce cell sensitivity to TNF (by shedding TNFR) while increasing soluble cytokine levels – contributing to a complex dysregulation of signaling. It also sheds epithelial and endothelial proteins that maintain barrier function. Thus ADAM17 is a key "hub" controlling availability of several mediators.	Soluble markers like sTNFR, sIL6R, and sTREM-1 in patient plasma reflect ADAM17 activity and correlate with severity (often rising in non-survivors). These could be used together as a proxy of ADAM17-driven immunopathology. In terms of therapy, inhibiting ADAM17 was found to **dampen inflammatory responses** in models (e.g. miR-145 targeting ADAM17 reduced sepsis inflammation). An ADAM17 inhibitor could simultaneously reduce TNF release and prevent shedding of receptors like IL-6R (which might keep IL-6 signaling localized rather than systemic). This multi-faceted intervention is novel and complex, aiming to restore homeostatic cell surface receptor landscapes during sepsis.

Sources: The information above is drawn from a broad literature base, including mechanistic studies and clinical investigations for each gene, as cited in-line (e.g.). These references illustrate the evidence supporting each gene's role and potential in sepsis. The comparative insights emphasize how leveraging these molecular insights can drive more tailored diagnostic and therapeutic strategies in sepsis management.

---

1 Distinguishing Sepsis From Infection by Neutrophil Dysfunction: A Promising Role of CXCR2 Surface Level - PMC
https://pmc.ncbi.nlm.nih.gov/articles/PMC7785795/

[2] [3] The Long Pentraxin PTX3 as a Humoral Innate Immunity Functional Player and Biomarker of Infections and Sepsis - PMC
https://pmc.ncbi.nlm.nih.gov/articles/PMC6473065/

[4] [6] [8] Frontiers | The role and therapeutic potential of SIRTs in sepsis
https://www.frontiersin.org/journals/immunology/articles/10.3389/fimmu.2024.1394925/full

[5] [7] Clinical significance of sirtuin 1 level in sepsis: correlation with disease risk, severity, and mortality risk - PMC
https://pmc.ncbi.nlm.nih.gov/articles/PMC7695447/

[9] Critical Care Explorations
https://journals.lww.com/ccejournal/fulltext/2021/11000/efficacy_and_safety_of_vilobelimab__ifx_1_,_a.18.aspx

[10] [11] [12] [13] [14] [16] [17] Therapeutic targeting of HMGB1 during experimental sepsis modulates the inflammatory cytokine profile to one associated with improved clinical outcomes | Scientific Reports
https://www.nature.com/articles/s41598-017-06205-z

[15] JCI - Angiopoietin 2 mediates microvascular and hemodynamic alterations in sepsis
https://www.jci.org/articles/view/66549
